# Genome-wide identification and characterization of abiotic stress responsive GRAS family genes in oat (*Avena sativa*)

**DOI:** 10.7717/peerj.15370

**Published:** 2023-05-09

**Authors:** Jing Pan, Qingping Zhou, Hui Wang, Youjun Chen, Zhiqiang Wang, Junchao Zhang

**Affiliations:** 1 Southwest Minzu University, Institute of Qinghai-Tibetan Plateau, Chengdu, Sichuan Province, China; 2 Southwest Minzu University, Sichuan Zoige Alpine Wetland Ecosystem National Observation and Research Station, Chengdu, Sichuan Province, China

**Keywords:** Oat, GRAS, Genome-wide, Expression pattern, Abiotic stress

## Abstract

**Background:**

GRAS transcription factors play a variety of functions in plant growth and development and are named after the first three transcription factors GAI (GIBBERRELLICACIDINSENSITIVE), RGA (REPRESSOROFGAI), and SCR (SCARECROW) found in this family. Oat (*Avena sativa*) is one of the most important forage grasses in the world. However, there are few reports on the GRAS gene family in oat.

**Methods:**

In order to understand the information and expression pattern of oat GRAS family members, we identified the GRAS members and analyzed their phylogenetic relationship, gene structure, and expression pattern in oat by bioinformatics technology.

**Results:**

The results showed that the oat GRAS family consists of 30 members, and most of the AsGRAS proteins were neutral or acidic proteins. The phylogenetic tree divided the oat GRAS members into four subfamilies, and each subfamily has different conservative domains and functions. Chromosome location analysis suggested that 30 *GRAS* genes were unevenly distributed on five chromosomes of oat. The results of real-time quantitative reverse transcription-PCR (qRT-PCR) showed that some *AsGRAS* genes (*AsGRAS12*, *AsGRAS14*, *AsGRAS21*, and *AsGRAS24*) were all up-regulated with increasing stress treatment time.The results of this study provide a theoretical basis for further research into the corresponding stress of oat. Therefore, further studies concentrating on these *AsGRAS* genes might reveal the many roles played by *GRAS* genes in oat.

## Introduction

A transcription factor (TF) is a protein that can directly or indirectly interact with *cis-*acting elements in the gene promoter region to regulate gene transcription. Transcription factors regulate plant growth and development and are composed of at least two structural domains: the DNA-binding domain and the transcription regulation domain (Riechmann et al., 2000). The GRAS gene family was named after the first three members of the gene family that were discovered: Gibberellic acid insensitive (GAI; Peng et al., 1997), Repressor of GA1 (RGA; Silverstone, Ciampaglio & Sun, 1998), and Scarecrow (SCR; Laurenzio et al., 1996). A typical GRAS protein is usually 400 to 770 amino acids in length and is mainly distributed in the nucleus (Phytochrome A signal transduction 1-PAT1 is distributed in the cytoplasm; Guo et al., 2017; Bolle, Koncz & Chua, 2000). GRAS proteins include a conserved C-terminus and a variable N-terminus. The C-terminus of the GRAS protein is highly conserved, indicating that it is critical to the functions of the protein. The N-terminus domain of the GRAS protein is variable, meaning that it may be involved in recognizing molecules during plant development (Sun et al., 2011).

GRAS proteins are widespread in plants and were initially divided into seven subfamilies based on the positions of their domain in *Arabidopsis*: SCR, DELLA, Ls (LATERAL SUPPRESSOR), SHR (SHORT ROOT), HAM (HAIRY MERISTEM), PAT1, and SCL9 (Bolle, 2004). Recent research has updated the division of the *Arabidopsis* GRAS transcription factors into eight subfamilies: DELLA, HAM, LISCL (*Lilium longiflourum* SCR-like), SCR, PAT1, Ls, SCL3, SHR (Tian et al., 2004). The release of genome-wide information for different plants has led to the GRAS family being classified slightly differently in other plants. Researchers compared and identified the GRAS protein sequences of *Arabidopsis*, rice, grape, and tomato, and divided the GRAS proteins into: DELLA, HAM, LISCL, AtSCR, AtPAT1, DLT, AtSCL3, AtSCL4/7, AtLAS, AtSHR, Pt20, Os19, Os4, and 13 other subfamilies (Liu & Widmer, 2014), with the GRAS family of model plants *Arabidopsis* and *Oryza sativa* containing eight subfamilies: DELLA, HAM, LISCL, PAT1 (Phytochrome A signal transduction), LS, SCR, SHR, and SCL3 (Tian et al., 2004). The number of GRAS subfamilies have ranged from eight to 17 in previous studies, with 12 identified in *Hordeum vulgare* (To et al., 2020), 10 in *Fagopyrum esculentum* (Liu et al., 2019), 16 in *Solanum lycopersicum* (Huang et al., 2015b), nine in *Glycine max* (Wang et al., 2020a), and 10 in *Cucumis sativus* (Lu et al., 2020).

The diverse structures of GRAS transcription factors have led to the assumption that these transcription factors play essential roles in plant growth and development, such as the radial elongation of embryos, root development, side branch formation, phytochrome signal transduction, meristem development, signal transduction by gibberellins, and stress response (Cenci & Rouard, 2017). The functions of GRAS members in different subfamilies are also distinct due to differences in the domains conserved in each subfamily. For example, members of the LISCL subfamily have been shown to regulate meiotic prophase in anthers and are involved in the formation of microspores in *Lily Microsporo-cytes* (Morohashi et al., 2003). SCL3 subfamily members help mediate root elongation (Heo et al., 2011). In addition, SCL3 proteins may interact with DELLA proteins to regulate gibberellin signal transduction *via* the IDD protein (Yoshida & Ueguchi-Tanaka, 2014). The DELLA subfamily members can negatively regulate the gibberellic acid (GA) signaling pathway as well as regulate plant growth and development (Keller et al., 2020). There is only one DELLA subfamily protein in *O. sativa*, SLR1, that stimulates GA signaling to relieve the interaction between SLR1 and to promote cellulose synthesis (Huang et al., 2015a). Members of the HAM subfamily are involved in meristem cell proliferation, chlorophyll synthesis, and transport among polar tissues (Li et al., 2003). GRAS proteins also play an important role in a plant’s response to stress. For example, high salt, low temperatures, and drought reduce the GA content in plants, resulting in the accumulation of DELLA subfamily proteins and the dwarfing of plants, which improves the plant’s stress tolerance. *Populus euphratica* GRAS gene *PeSCL7* is induced by drought and high-salt stress (Ma, Xia & Yin, 2011); high NaCl treatment of *Halostachys caspica* seedlings caused the expression of GRAS family gene *HcSCL13* to be gradually up-regulated with time, indicating that *HcSCL13* could respond positively to salt stress (Zhou et al., 2013).

Oat (*Avena sativa* L.) is an annual cereal and fodder crop of the Poaceae Barnhart family that is grown in approximately 42 countries around the world (Bräutigam et al., 2005). Oat is also widely cultivated in the North China Plain, Northwest Plateau, and Northeast China due to its high-stress resistance and feed value (Liu et al., 2007). With the release of genomic data of the oat, researchers can now perform extensive studies on the molecular mechanisms of growth, development, and stress regulation in oat at a genomic level. The role of GRAS transcription factors in oat growth and development has not yet been identified and analyzed on a genome-wide scale. Because of the role of *GRAS* genes in plant growth and development, the systematic identification of these genes in oat is important for understanding the mechanisms behind the growth and development of oat. This study aimed to use bioinformatics technology to identify GRAS family genes present in oat on a genome-wide scale and to explore their gene structures, protein structures, and genetic relationships. This work will provide data for the further elucidation of the molecular mechanisms of growth and the developmental regulation of the oat *GRAS* genes.

## Materials and Methods

### Identification and sequence analysis of *GRAS* genes in oat

To identify the *GRAS* genes present in oat, the first version of the whole genome sequence of oat (DNA and Gff3) was downloaded from the results of the joint sequencing study done by PepsiCo and Corteva Agriscience (https://wheat.pw.usda.gov/GG3/graingenes_downloads/oat-ot3098-pepsico). The protein sequences of *A. thaliana* were downloaded from the TAIR database (https://www.Arabidopsis.org/). The Hidden Markov Model (HMM) file of the GRAS domain (PF03514) was downloaded from the Pfam protein family database (http://pfam.sanger.ac.uk/). The GRAS protein sequences of oat were aligned using the HMM model in HMMER 3.1 software (National Institutes of Health, Bethesda, MD, USA). We extracted the GRAS family member sequences of oat using the TBtools software (Chen et al., 2020a). The resulting protein sequence of GRAS was verified using the SMART (http://smart.embl.de/) and PFAM (http://pfam.xfam.org/) programs. The protein sequence of AsGRAS was further verified using the NCBI protein database (https://blast.ncbi.nlm.nih.gov/Blast.cgi), BLASTp.

### Phylogenetic analysis of *GRAS* genes in oat

To further investigate the phylogeny of the oat *GRAS* genes, we created a phylogenetic tree using the neighbour-joining (NJ) method. A multiple sequence alignment was performed and a phylogenetic tree of the *Arabidopsis* and oat *GRAS* gene families was constructed using the MEGA-X software (https://www.megasoftware.net/; Kumar et al., 2018). The NJ phylogenetic tree was built with 1,000 bootstrap replications. All the identified *AsGRAS* genes were then divided into different clusters based on AsGRAS classification. The GRAS protein sequences (*A. thaliana*, *O. sativa*, *Z. mays*) were downloaded from the *Ensembl* database (http://ensemblgenomes.org/). ITOL (https://itol.embl.de/) and Adobe Photoshop 2019 (https://www.adobe.com/products/photoshop.html?promoid=RBS7NL7F&mv=other) were used to depict the modified phylogenetic tree.

### Analysis of conserved motifs of oat

The exons, coding sequences, and untranslated regions on the oat chromosome were extracted from the gff3 file and the TBtools software was used to map the structure of the gene (Chen et al., 2020a). A multiple sequence alignment of AsGRAS proteins was performed using the Jalview software package (Version 2.10.5), with the parameters of the alignment process set to default values, and the results color-coded by the BioEdit software (Hall, 2011). The sequences of the conserved proteins in AsGRAS were identified using the MEME Suit online tool (Bailey et al., 2009; Version 5.3.3, https://meme-suite.org/meme/tools/meme) and visualized using the TBtools software. The physicochemical properties of GRAS proteins in oat were predicted using the ExPASy proteomics server, and their subcellular localizations were identified using the Cell-PLoc online software (Chou & Shen, 2010).

### Chromosomal location and homology analysis of *GRAS* genes in oat

The CDS and gene sequences corresponding to all *GRAS* genes in oat were retrieved from the oat genome files using the TBtools tool, and the introns of the *AsGRAS* genes were analyzed using the GSDS 2.0 online website (gene structure display server, http://gsds.gao-lab.org/) and the exon structure was used for predictive analyses. The corresponding positions of the AsGRAS genes on the chromosome were identified using the GFF3 data from the oat genome (Chen et al., 2020b), and TBtools (Chen et al., 2020a) was used to complete the visualization of the location of the genes on the chromosome. In order to explore the *GRAS* gene homology between oat and other species, we downloaded genome-wide data and the gene annotation files for *Arabidopsis* (*Arabidopsis thaliana*), rice (*O. sativa*), maize (*Zea mays*) wheat (*Triticum aestivum*), barley (*Hordeum vulgare*), foxtail (*Setaria viridis*), teff (*Eragrostis curvula*), and millet (*Setaria italica*) from the online website EnsemblPlants (http://ensemblgenomes.org/). We then performed an analysis of *AsGRAS* gene replication events using the “One-Step MCscanX” function in the TBtools software. The “Dual Synteny Plot” function in the TBtools software was used to build the multispecies collinearity analysis table, and the nonsynonymous substitution value (Ka) and synonymous substitution value (Ks) of the GRAS repeat gene were then calculated.

### Analysis of *cis-*acting elements in the AsGRAS gene family

The 2,000 bp upstream base of the start codon of the oat GRAS gene family was used as the sequence in the PLACE (http://www.dna.affrc.go.jp/PLACE/signalscan) online software. The TBtools (Chen et al., 2020a) software was then used to analyze the sequence with the extracted elements used to perform predictive analysis on the *cis*-acting elements of this gene family. The *cis-*elements of the AsGRAS gene family were visualized by the “Simple BioSequence Viewer” function of the TBtools software.

### Plant material and stress treatments

The oat variety selected in this experiment was “Qinghai 444,” and the seeds were provided by the Germplasm Restoration Laboratory of Qinghai-Tibet Plateau Research Institute, Southwest Minzu University. This experiment began on September 25, 2021 with full-grained seeds soaked in 75% ethanol for 1 min for disinfection, and then germinated on filter paper for about 48 h. The oat seedlings with consistent growth were then transferred to 1/2 Hoagland nutrient solution (PH = 5.8) in 16 h light/8 h darkness with a light intensity of about 8,000 lx. The seedlings were then grown and cultured at 28 °C until the two-leaf stage, and the nutrient solution was replaced every 2 days. The oat plants with relatively consistent growth for 3 weeks were selected to start drought stress (20% PEG-6000; Macklin, Dunmurry, UK) and salt stress (0.9% NaCl; Macklin, Dunmurry, UK) treatments (Wang et al., 2020c). The treatment time points were set at 1, 3, 6, 24, and 48 h, with one control (0 h). The second leaf from the top of each plant and a root sample from each plant were then taken and immersed in liquid nitrogen for quick freezing and then stored in a −80 °C freezer. Three independent biological replicates were performed, each with 3–6 individual plants.

### Expression analysis of the *AsGRAS* genes by real-time PCR

Total RNA was extracted from the root and leaf tissues of oat plants under different treatment schedules using the MiniBEST Plant RNA Extraction Kit (Takara, Kusatsu, Japan), and reverse transcription was carried out using a cDNA synthesis kit (PrimeScriptTM RT reagent Kit with gDNA Eraser; Takara, Kusatsu, Japan). The cDNA was removed after the completion of the genome, and cDNA concentration was detected using a NanoDrop 2000 UV spectrophotometer (Thermo Fisher Scientific, Waltham, MA, USA) and then diluted uniformly to a concentration of 50 ng·μL-1 for the qRT-PCR reaction matrix. Specific primers for analysis were designed with Primer software (Version 5.0; Table S1) and the qRT-PCR analysis was performed using the StepOne Fast Real-Time PCR System (applied biosystems, Waltham, MA, USA) with three technical replicates. The amplification reaction system contained 2 µL of diluted cDNA solution, 1 µL of forward and reverse primers, 12.5 µL of SYBR Premix Ex Taq II (Japanese, Takara, Kusatsu, Japan), and 8.5 µL of sterile water for a total volume of 20 µL. The reaction program was: denaturation at 95 °C for 30 s, denaturation at 95 °C for 5 s, annealing at 60 °C for 30 s, for 40 cycles, followed by 95 °C for 15 s, 60 °C for 30 s, and 95 °C for 15 s. Using GAPDH (Tajti, Pál & Janda, 2021) as the internal reference gene, each sample was replicated three times, and the relative expression was calculated using the 2^−ΔΔCt^ method (Livak & Schmittgen, 2001).

### Statistical analysis

All data in this study were analyzed using the Origin 2020 pro statistical analysis program (OriginLab Corporation, Northampton, MA, USA) using analysis of variance, and means were compared using least significant difference tests (LSD) at the 0.05 level of significance.

## Results

### Identification of GRAS family numbers in oat

In this study, 30 GRAS proteins were identified in oat and were named AsGRAS01 to AsGRAS30 according to their physical location on the chromosome (Table S2). In this study, we analyzed the basic characteristics of these 30 AsGRAS proteins, including their coding sequence length (CDS), protein molecular weight (MW), isoelectric point (pI), and chromosomal location (Location; Table 1). Among these 30 AsGRAS proteins, AsGRAS24 was the shortest with only 291 amino acids, and AsGRAS30 was the largest with 635 amino acids. The molecular weight of the proteins ranged between 22.27 kDa (AsGRAS09) and 66.62 kDa (AsGRAS25). The pI value of the proteins ranged between 5.38 (AsGRAS17) and 9.50 (AsGRAS09), with a mean pI value of 6.01, indicating that the oat GRAS protein was acidic. More than half of the AsGRAS proteins were distributed on three chromosomes: chromosome 1A (*n* = 12, 40%), chromosome 1D (*n* = 7, 23.3%), and chromosome 2C (*n* = 5, 16%), and chromosome 1C had only two genes (6%).

**Table 1:** Information on the *GRAS* genes identified in oat.

Gene name	ID	Chromosome location	Amino acid length	pI	Molecular weight (kDa)	Subcellular localization
*AsGRAS01*	Pepsico1_Contig29889.mrna1	1A	617	5.77	65.3	Nucleus
*AsGRAS02*	Pepsico2_Contig16967.mrna1	1A	496	5.84	52.91	Nucleus
*AsGRAS03*	Pepsico2_Contig1709.mrna2	1A	541	5.37	59.86	Nucleus
*AsGRAS04*	Pepsico1_Contig26725.mrna2	1A	615	5.73	65.07	Nucleus
*AsGRAS05*	Pepsico1_Contig13965.mrna2	1A	615	5.73	65.07	Nucleus
*AsGRAS06*	Pepsico1_Contig30502.mrna2	1A	541	5.37	59.86	Nucleus
*AsGRAS07*	Pepsico1_Contig18352.mrna2	1A	541	5.37	59.86	Nucleus
*AsGRAS08*	Pepsico1_Contig25100.mrna1	1A	617	5.77	65.3	Nucleus
*AsGRAS09*	Pepsico2_Contig4966.mrna2	1A	216	9.5	22.27	Nucleus
*AsGRAS10*	Pepsico2_Contig7359.mrna1	1A	617	5.77	65.3	Nucleus
*AsGRAS11*	Pepsico1_Contig19030.mrna1	1A	617	5.77	65.3	Nucleus
*AsGRAS12*	Pepsico1_Contig14278.mrna1	1A	617	5.77	65.3	Nucleus
*AsGRAS13*	Pepsico1_Contig14835.mrna1	1C	617	5.79	65.31	Nucleus
*AsGRAS14*	Pepsico1_Contig27727.mrna1	1C	617	5.79	65.31	Nucleus
*AsGRAS15*	Pepsico1_Contig30502.mrna1	1D	541	5.38	59.95	Nucleus
*AsGRAS16*	Pepsico2_Contig7359.mrna2	1D	615	5.76	65.08	Nucleus
*AsGRAS17*	Pepsico1_Contig18352.mrna1	1D	541	5.38	59.95	Nucleus
*AsGRAS18*	Pepsico2_Contig16943.mrna3	1D	538	6.17	60.3	Nucleus
*AsGRAS19*	Pepsico2_Contig5101.mrna1	1D	538	6.17	60.3	Nucleus
*AsGRAS20*	Pepsico2_Contig1709.mrna1	1D	541	5.38	59.95	Nucleus
*AsGRAS21*	Pepsico1_Contig29889.mrna2	1D	615	5.76	65.08	Nucleus
*AsGRAS22*	Pepsico1_Contig17925.mrna1	2C	319	6.1	35.5	Nucleus
*AsGRAS23*	Pepsico2_Contig10379.mrna3	2C	571	6.02	64.4	Nucleus
*AsGRAS24*	Pepsico1_Contig36247.mrna1	2C	291	7.1	32.44	Nucleus
*AsGRAS25*	Pepsico1_Contig17438.mrna1	2C	634	6.91	66.62	Nucleus
*AsGRAS26*	Pepsico2_Contig1385.mrna1	2C	571	6.02	64.4	Nucleus
*AsGRAS27*	Pepsico2_Contig8490.mrna1	2D	461	5.87	49.88	Nucleus
*AsGRAS28*	Pepsico2_Contig1385.mrna2	2D	571	6.06	64.27	Nucleus
*AsGRAS29*	Pepsico2_Contig10379.mrna1	2D	571	6.06	64.27	Nucleus
*AsGRAS30*	Pepsico1_Contig28179.mrna1	2D	635	6.69	66.54	Nucleus

### Phylogenetic analysis and classification of *AsGRAS* genes

**Figure 1:**
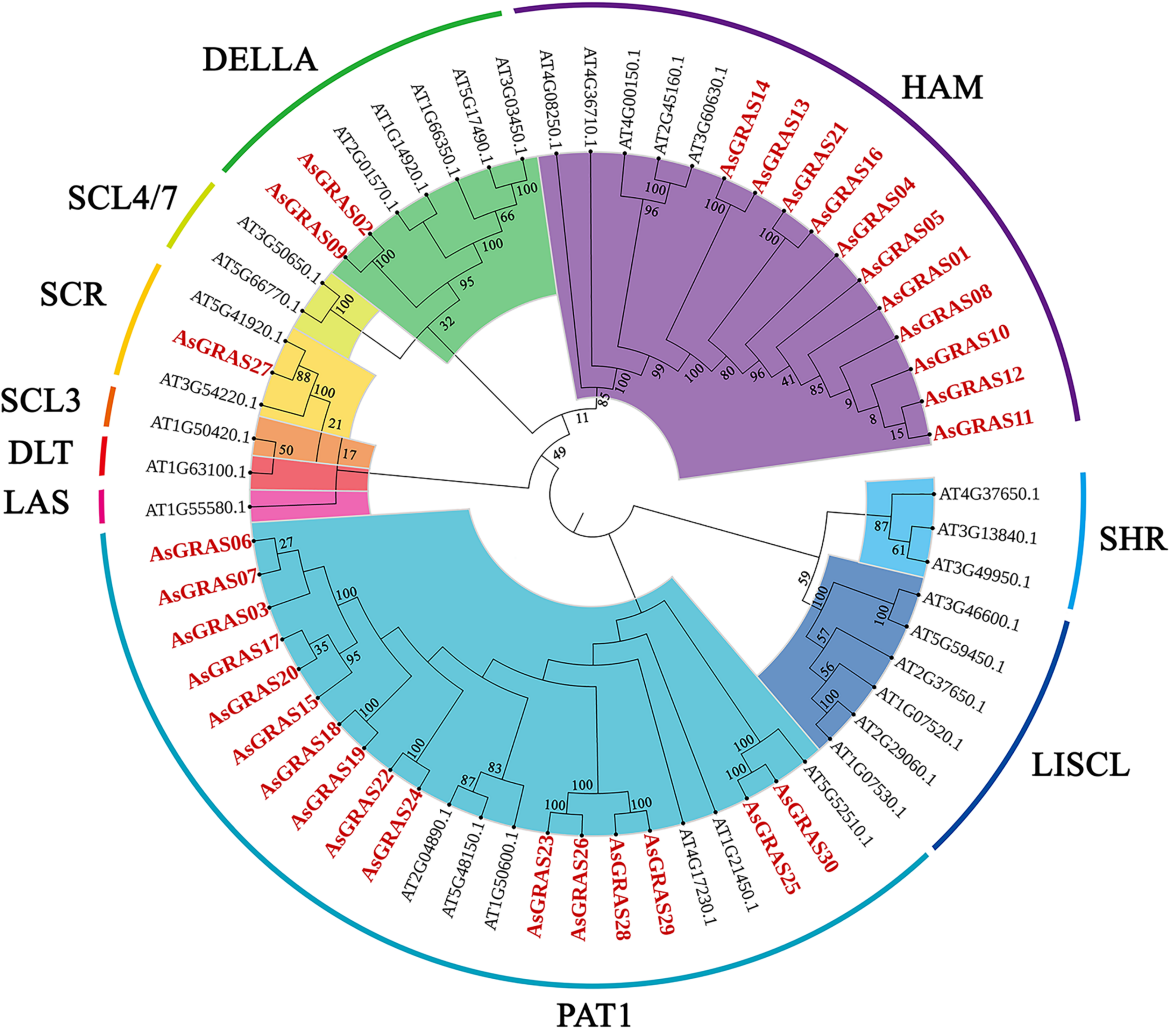
Phylogenetic tree of GRAS proteins in *Avena sativa* and *Arabidopsis*. The GRAS protein sequences of the two species were aligned by MEGA X using the MUSCLE method. The phylogenetic tree was built using the neighbor-joining (NJ) method and further categorized into ten distinct subfamilies, shown in different colors. All the AsGRAS proteins have been emphasized in red.

To explore the phylogenetic relationship of the GRAS proteins identified in oat, we constructed a phylogenetic tree based on the amino acid sequences of the 30 AsGRAS proteins (Table S3) and 32 AtGRAS proteins using the neighbor-joining (NJ) method (Fig. 1). According to their homology to *Arabidopsis* GRAS proteins, the 30 GRAS proteins of oat were divided into four subfamilies: SCR, PAT1, HAM, and DELLA. The PAT1 subfamily was the largest, with 16 *AsGRAS* genes. The SCR subfamily contained only one *AsGRAS* gene, and the HAM and DELLA subfamilies had 11 and two *AsGRAS* genes, respectively.

**Figure 2:**
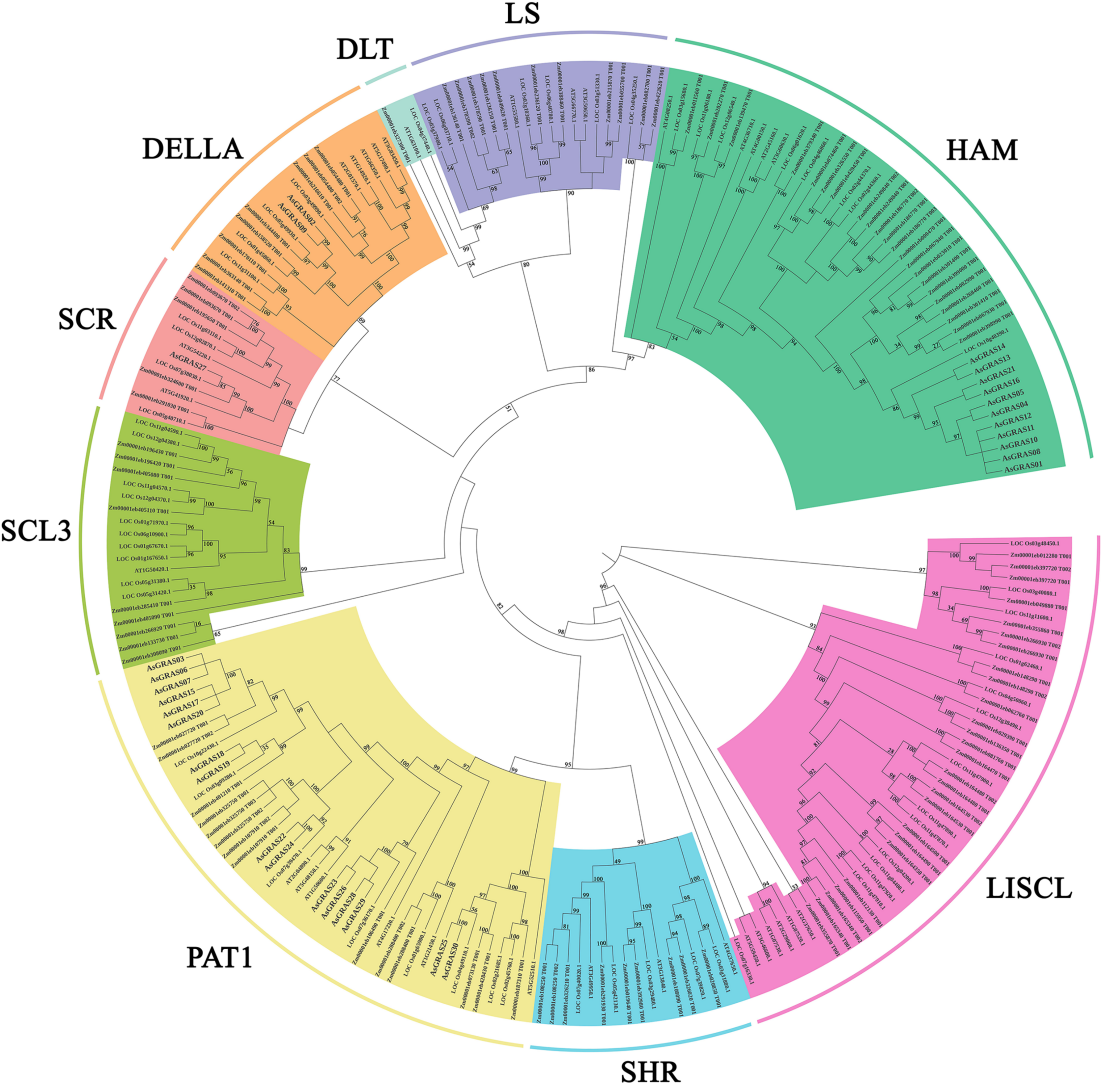
Phylogenetic tree of GRAS proteins from *Avena sativa, A. thaliana, O. sativa*, and *Z. mays*. The GRAS protein sequences of the four species were aligned by MEGA X using the MUSCLE method. The phylogenetic tree was built using the neighbor-joining (NJ) method and further categorized into nine distinct subfamilies, shown in different colors.

This study further investigated the diversity of the evolutionary process of the GRAS family of genes in oat by analyzing 32 *Arabidopsis GRAS* genes, 60 *O. sativa GRAS* genes, 105 *Z. mays GRAS* genes, and 30 *A. sativa GRAS* genes for genetic relationships (Table S4). According to branching angle, developmental tree topology, and *Arabidopsis* classification, 227 *GRAS* genes were divided into nine subfamilies (Fig. 2). The LISCL and HAM subfamilies contained the most *GRAS* genes, while the LAS and SCL4/7 subfamilies had the fewest. The LAS and SCL4/7 subfamilies were not recognized in oat, indicating that these subfamilies occur in oat as a species with specific deletions.

### Gene structure and motif composition of the *AsGRAS* gene family

**Figure 3:**
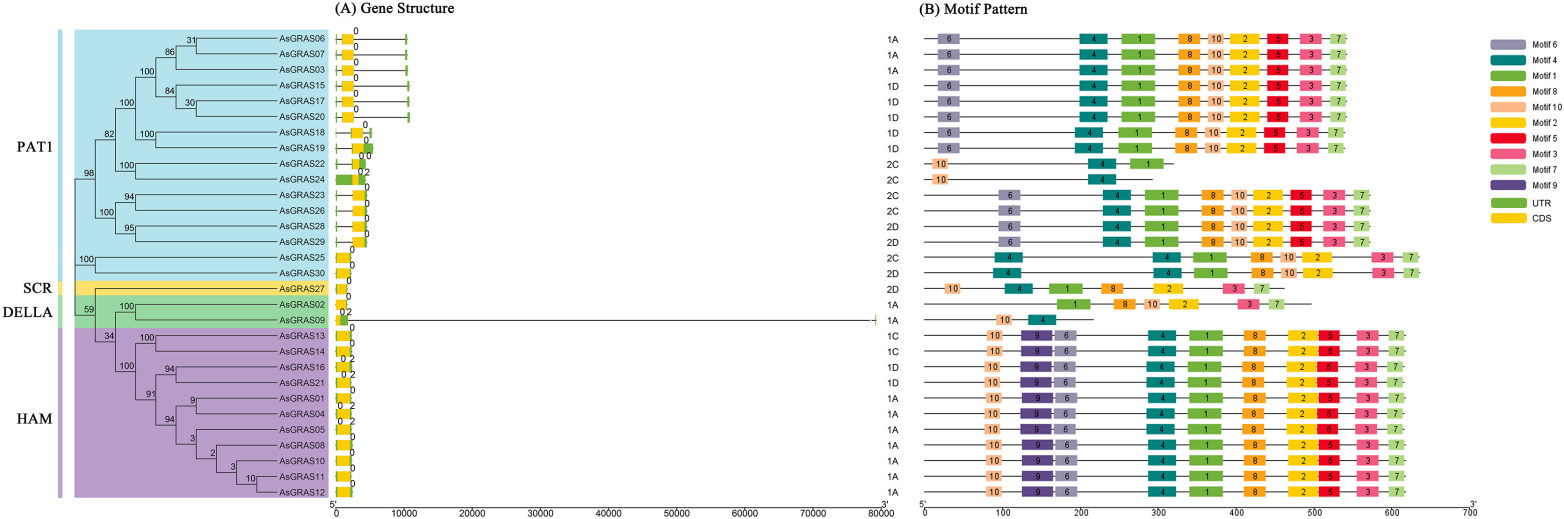
Structural analysis of *AsGRAS* genes in *Avena sativa*. (A) The exon-intron structure of *GRAS* genes. (B) The distribution of motifs in GRAS proteins.

The genomic DNA sequences of the *AsGRAS* gene were used to create exon and intron structure diagrams of the *AsGRAS* gene to better understand its structural composition (Fig. 3A). All 30 *AsGRAS* genes contained a GRAS domain, and most (24, 80%) contained no introns. *AsGRAS04*, *AsGRAS05*, *AsGRAS09*, *AsGRAS16*, *AsGRAS21*, and *AsGRAS24* each contained two introns. In general, members of the same subfamily had similar gene structures. The protein conserved sequences and sequence logos of the *GRAS* gene family members in oat were analyzed using MEME online analysis software. A total of 10 conserved motifs (named motif 1–motif 10) were identified, and more motifs were located at the C-terminus than at the N-terminus (Fig. 3B). Most AsGRAS proteins contained motif 4 and motif 10. *AsGRAS02* did not have motif 4, while *AsGRAS09* only included motifs 4 and 10. Some motifs were only present in specific subfamilies, for example, motif 9 was only present in the HAM subfamily. A comparison of the members of the *AsGRAS* gene family found that the type, number, and sequence of protein motifs of members of the same subfamily were remarkably conserved. Most closely related members had similar motifs: for example, the PAT1 group included motif 6, motif 4, motif 1, motif 8, motif 2, motif 5, motif 3, and motif 7, while the SCR group contained motif 10, motif 4, motif 1, motif 8, motif 2, motif 3, and motif 7.

### Chromosomal distribution and collinearity analysis of *AsGRAS* genes

**Figure 4:**
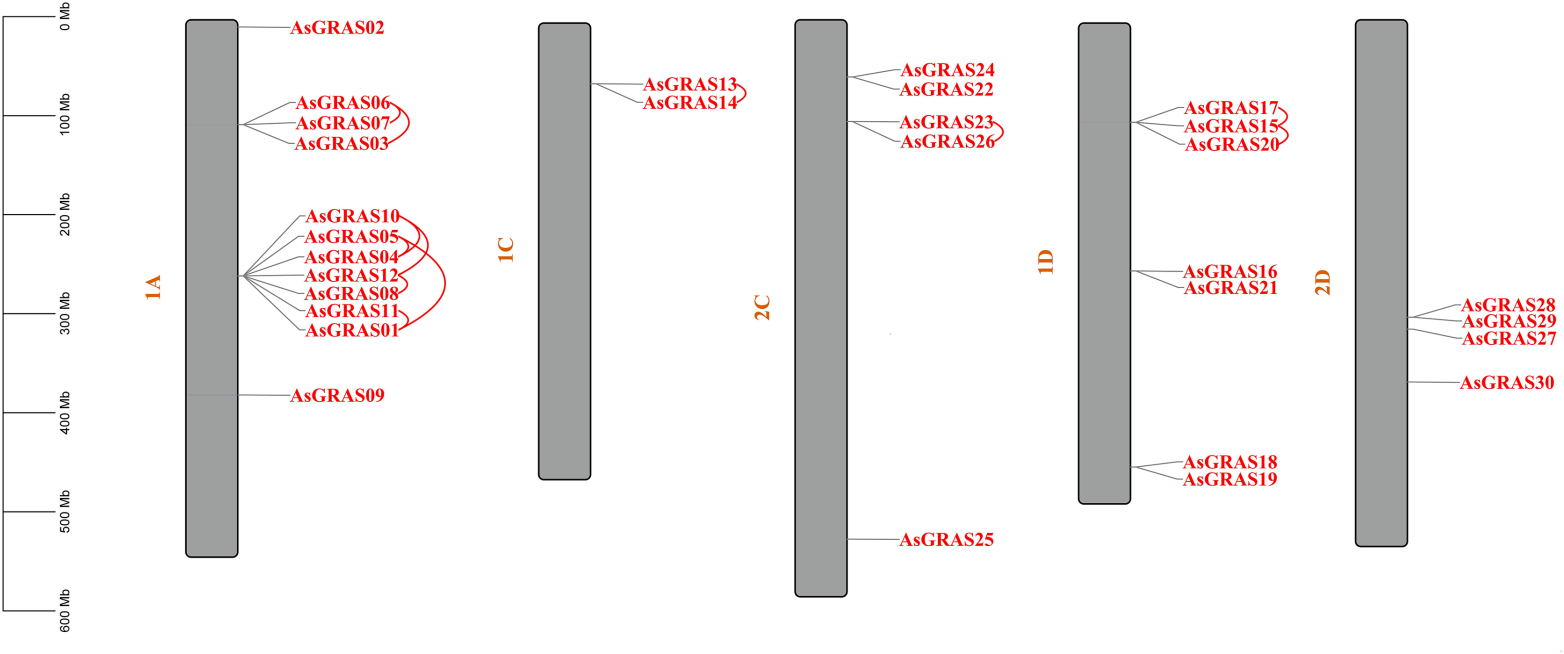
Schematic representations of the chromosomal distribution of oat *GRAS* genes. The red lines indicate duplicated *GRAS* gene pairs.

**Figure 5:**
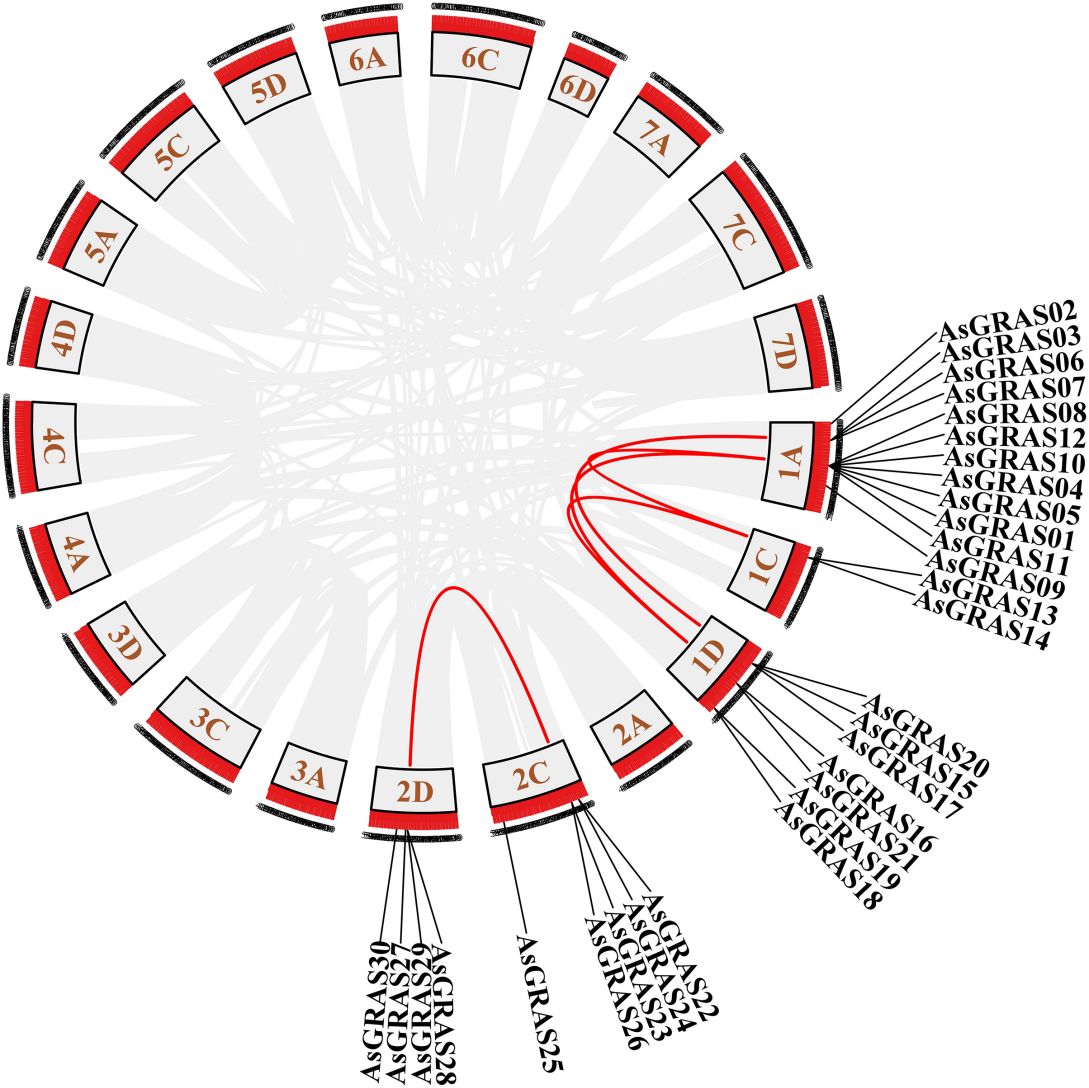
Chromosome localization of duplicated *AsGRAS* genes in *Avena sativa*. The red lines represent the segmentally duplicated genes and the black bands represent the collinear block.

The distribution of *GRAS* genes on the oat chromosomes were also analyzed using the oat genome (Fig. 4). The results showed that the 30 *AsGRAS* genes were unevenly distributed on the five chromosomes in oat with chromosome 1A having the most genes (12, 40%), followed by chromosome 1D (7, accounting for about 23.3%), chromosome 2C (5, about 16.7%), chromosome 2D (4, about 13.3%), and chromosome 1C (2, about 6.7%). In this study, 17 *AsGRAS* genes were clustered into 12 tandem duplication event regions in oat chromosomes 1A, 1C, 2C, and 1D (Fig. 5), indicating that these regions were hot spots of *AsGRAS* gene distribution. Chromosome 1A had eight clusters (*AsGRAS06*/*AsGRAS07*, *AsGRAS06*/*AsGRAS03*, *AsGRAS10*/*AsGRAS04*, *AsGRAS10*/*AsGRAS12*, *AsGRAS05*/*AsGRAS04*, *AsGRAS05*/*AsGRAS01*, *AsGRAS12*/*AsGRAS08*, *AsGRAS11*/*AsGRAS01*), Chromosome 1C had one cluster (*AsGRAS13*/*AsGRAS14*), Chromosome 2C also had one cluster (*AsGRAS23*/*AsGRAS26*), and Chromosome 1D had two clusters (*AsGRAS17*/*AsGRAS15*, *AsGRAS15*/*AsGRAS20*). This suggests an evolutionary relationship between these AsGRAS members as these genes all had similar structures and functions, and originated from chromosomal duplication. The collinear analysis of AsGRAS proteins showed that the genes with collinear relationships were located in the same subfamily.

**Figure 6:**
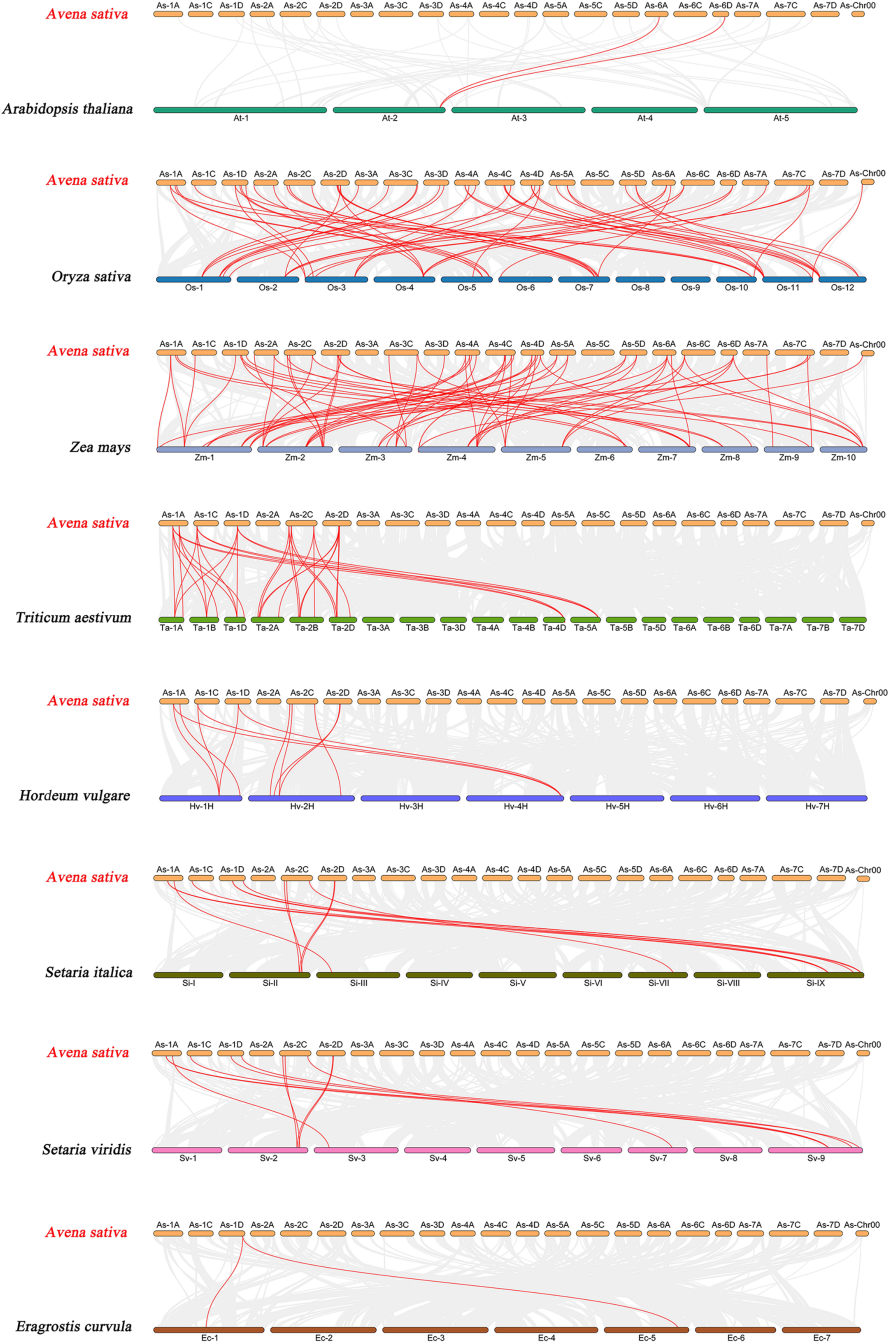
Synteny analyses of the *GRAS* genes between *Avena sativa* and eight representative species. The gray lines indicate the collinear blocks within *A. sativa* and other species genomes. The syntenic *GRAS* gene pairs between oat and other species are highlighted with red lines.

To further explore the evolutionary clues of the oat GRAS gene family, this study constructed eight representative comparative syntenic graphs of GRAS family members between oat and eight representative species (Fig. 6). The eight species included one dicot (*A. thaliana*) and seven monocots (*Z. mays*, *O. sativa*, *T. aestivum*, *H. vulgare*, *Setaria italica*, *Setaria viridis*, and *Eragrostis curvula*). A total of 30 *AsGRAS* gene members were associated with *Arabidopsis* (30), *O. sativa* (30), *Z. mays* (30), *T. aestivum* (30), *H. vulgare* (30), *S. italica* (29), *S. viridis* (30), and the genes of *E. curvula* (30) had syntenic relationships (Table S5). A total of 70, 57, 33, 12, 12, 11, and 2 orthologous AsGRAS gene pairs were identified between oat and *Z. mays*, *O. sativa*, *T. aestivum*, *H. vulgare*, *S. italica*, *S. viridis*, *E. curvula*, and *A. thaliana*, respectively. In general, *AsGRAS* genes consisted of more monocotyledonous symbiotic gene pairs. The analysis of collinearity between oat and other species have important implications for elucidating the evolution of *GRAS* genes.

### *Cis*-acting element analysis of *AsGRAS* genes

**Figure 7:**
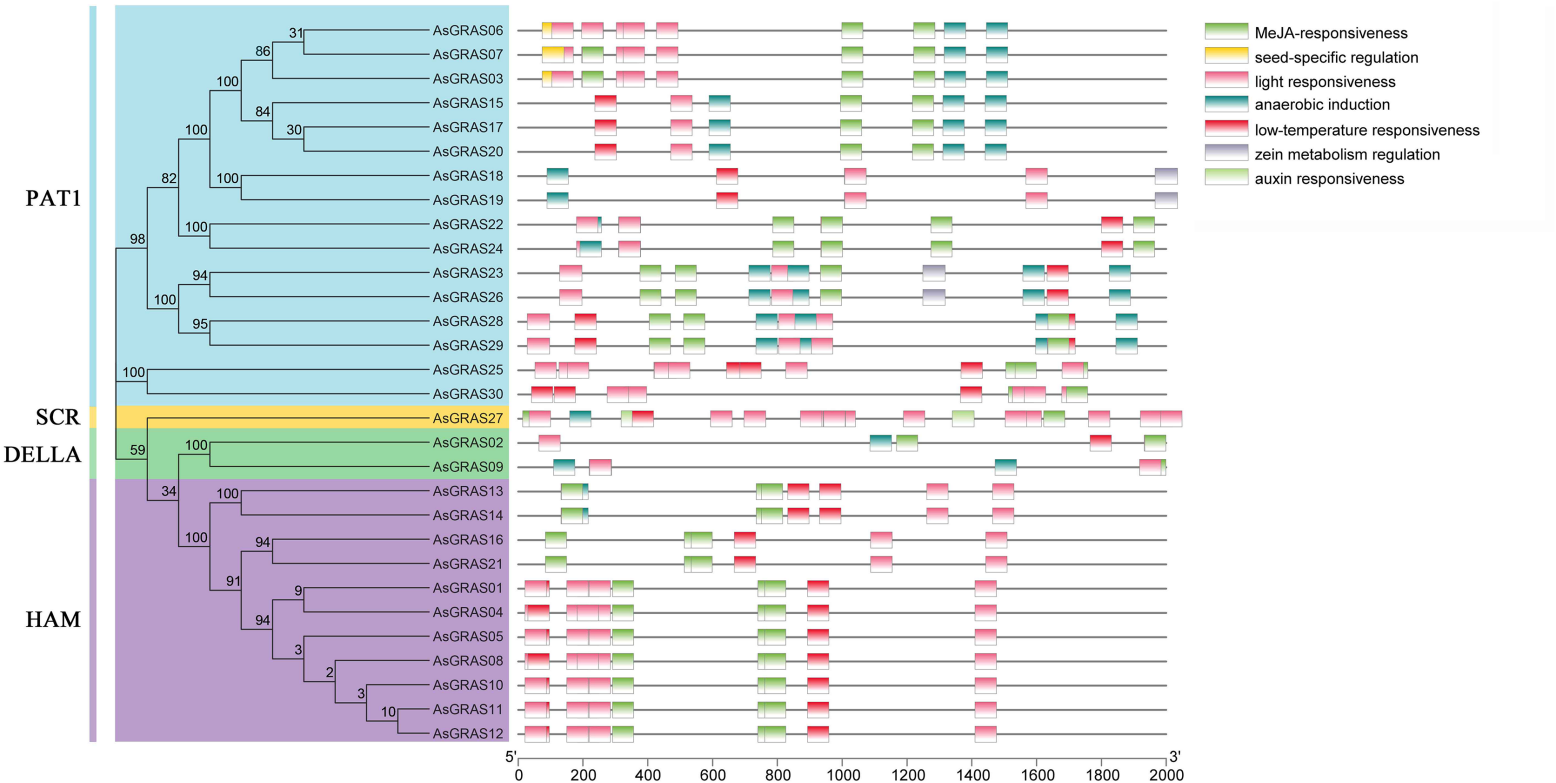
*Cis*-acting elements in promoters of AsGRAS family members.

*Cis*-acting elements are specific binding sites for proteins involved in transcription initiation and regulation and play a role in plant gene transcription regulation (Hernandez-Garcia & Finer, 2014). To determine the *cis-*acting elements in the AsGRAS promoter region, the *cis-*element types and potential functions of the oat GRAS gene family were analyzed using PLACE online software (http://bioinformatics.psb.ugent.be/webtools/plantcare/html/). The results showed (Fig. 7 and Table S6) that there were a large number of *cis-*regulatory elements in the promoter region of the oat GRAS gene family, including light response element, abscisic acid response element, low-temperature stress response element, drought stress response *cis-*acting elements, and tissue-specific expression elements. The *cis-*acting elements contained in the promoter regions of different members of the AsGRAS gene family had similarities and differences in both types and number of elements. For example, all *AsGRAS* genes contained light-responsive elements, and most of the *AsGRAS* genes contained abscisic acid-responsive elements (26) and low-temperature stress response elements (26). Some *AsGRAS* genes contained drought-responsive elements (20). The *AsGRAS27* gene contained more *cis-*acting elements (49), while the *AsGRAS02* (11) and *AsGRAS09* (9) genes of the DELLA subfamily contained fewer *cis-*acting elements. The analysis results showed that *AsGRAS* genes play an essential regulatory role in different stress-resistant habitats.

### Expression of *AsGRAS* genes under salt stress and drought stress

**Figure 8:**
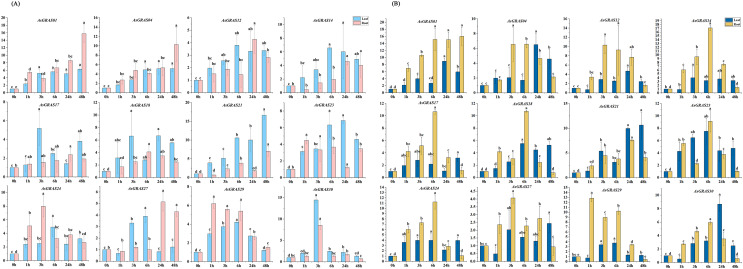
(A) The expression patterns of 12 *AsGRAS* genes were analyzed under drought stress by real-time quantitative RT-PCR. (B) The expression patterns of 12 *AsGRAS* genes were analyzed under salt stress by real-time quantitative RT-PCR. The relative expression levels, normalized to GAPDH, were determined by the comparative CT method (2^−ΔΔCT^). Three biological replicates were conducted for each experiment (Tukey’s multiple range tests, *P* < 0.05).

With the aim of further investigating the expression level of *AsGRAS* genes under different stresses and their potential functions in the developmental process, 12 *AsGRAS* genes were selected from the identified *GRAS* genes for qRT-PCR experiment verification in oat. Each AsGRAS gene subfamily is shown in Fig. 8 and Table S2, and the specific primers used for the qRT-PCR analysis are shown in Table S1. Significant changes in *AsGRAS* gene expression patterns were observed in two plant tissues (leaf and root), and some of these genes exhibited significant expression in the plant tissues. Results from the qRT-PCR experiments indicated that the selected genes often exhibited different expression profiles in the different *AsGRAS* gene subfamilies. However, selected genes in some subfamilies showed similar expression patterns. For example, under salt stress, the expression levels of *AsGRAS04* and *AsGRAS12* of the HAM subfamily were upregulated in the leaf tissue from 0-HAS (hour after stress) to 3-HAS and then down-regulated in a stepwise manner starting at 6-HAS. There were also different patterns of gene expression between members of the same subfamily. For instance, under osmotic stress, the expression of *AsGRAS24* of the PAT1 subfamily was upregulated in leaf tissue from 0-HAS to 6-HAS and then gradually decreased from 24-HAS onwards. In contrast, *AsGRAS30* was upregulated from 0-HAS to 3-HAS in leaf tissue and then gradually down-regulated from 6-HAS. These results indicate that the selection of representative *GRAS* gene expression profiles in oat can describe different *AsGRAS* gene expression patterns, underscoring the regulation of *AsGRAS* genes in the process of oat growth.

## Discussion

The GRAS gene family is a unique transcription factor family in plants and plays an essential role in plant growth, photosynthesis, development, and reproduction (Bolle, Koncz & Chua, 2000). Earlier studies showed that the GRAS family of genes evolved from prokaryotes to land plants through gene duplication (Zhang, Iyer & Aravind, 2012), and our analysis of the oat GRAS gene structure found that most of the AsGRAS genes (24, 80%) did not contain introns. This high percentage of intron-free *GRAS* genes is comparable to *Fagopyrum tataricum* (87%; Liu et al., 2019), *C. sativus* (54%; Lu et al., 2020), *Dactylis glomerata* (57%; Xu et al., 2020) and *S. lycopersicum* (77%; Huang et al., 2015b). Increased gene sequencing has led to abundant evidence that each eukaryotic ancestor had genes rich in introns, and that the majority of eukaryotes have experienced intron loss during species evolution (William Roy & Gilbert, 2006). The proportion of genes without introns was higher in some plants (*A. sativa*, *F. tataricum*, *C. sativus*, *D. glomerata*, and *S. lycopersicum*), indicating that these plants likely experienced intron loss as part of their evolutionary process. Initial studies indicated that gene duplication events played an essential role in the generation of novel functions and in the expansion of gene families, and chromosomal regions with two or more genes less than 200 kb were defined as tandemly duplicated events (Holub, 2001). Consistent with studies of *GRAS* genes in other species, there was no apparent correlation between the length of the chromosome and the number of *AsGRAS* genes in this study (Liu et al., 2019). Most transcription factors have domains and motifs that regulate protein interactions and the modification of DNA binding activity (Liu, White & MacRae, 1999). In this study, ten conserved motifs were identified in oat. Most *AsGRAS* genes contained all of the conserved motifs, and the species, number, and sequence of conserved protein motifs encoded by members of the same subfamily were found to be relatively constant. For example, the PAT1 subfamily contained motif 6, motif 4, motif 1, motif 8, motif 2, motif 5, motif 3, and motif 7, while the SCR subfamily contained motif 10, motif 4, motif 1, motif 8, motif 2, motif 3, and motif 7.

With the publication of genome sequencing data from different plant species in recent years, the genome-wide identification of members of the GRAS gene family has also been done for different plant species, including *O. sativa* (Tian et al., 2004), *T. aestivum* (Kumar & Bhalothia, 2021), *H. vulgare* (To et al., 2020), *G. max* (Wang et al., 2020b), and *C. sativus* (Lu et al., 2020). Oat is an annual grass crop that is widely used because of its strong stress resistance and high nutritional value (Zhang et al., 2021a). However, there has not yet been a study on *GRAS* genes at the whole genome level in oat. In this study, the GRAS genes present in oat plants were systematically analyzed using bioinformatics technology based on the published whole genome information of oat. Thirty members of the GRAS gene family were identified from the oat genome in this study. There were fewer members of the GRAS gene family in oat than in plants like *O. sativa* (62), *Z. mays* (105), *H. vulgare* (62), *G. max* (117), and *C. sativus* (37); these differences may be related to gene duplication and genome size (Zhang, Iyer & Aravind, 2012). Therefore, we divided the 30 oat GRAS proteins into four subfamilies: DELLA, PAT1, HAM, and SCR, according to their dendritic branching topology in *Arabidopsis*. Many studies have summarized the functional diversity of plant *GRAS* genes. For instance, the DELLA subfamily is related to light signal, abiotic stress, gibberellin signal, and ethylene signal transduction (Phokas & Coates, 2021), and the PAT1 subfamily is related to auxin signaling, phytochrome A signaling, and abiotic stress (Zuo et al., 2021). The HAM subfamily is related to the maintenance of the shoot apical meristem and auxin signaling (Han et al., 2020), and the SCR subfamily is involved in the development of bundle sheath cells and is associated with leaf development (Lee et al., 2008). We hypothesized that some key *AsGRAS* genes played a role in regulating plant growth and development. To explore the evolutionary relationship of GRAS family genes among different species, we performed a homology analysis on seven monocots and one dicot. The results showed that oat and *O. sativa* showed the best syntenic relationship. The results also showed that species with syntenic relationships to the oat *GRAS* genes nearly all derive from grasses such as rice and maize, indicating that the AsGRAS family had evolutionary conservation as well as a relatively close homology to related species. In contrast, *A. sativa*, *E. curvula*, and *Arabidopsis* showed the weakest connections. *GRAS* genes in monocots exhibited better syntenic relationships to the identified *AsGRAS* genes. We thus hypothesized that the collinear relationship observed among *GRAS* genes was related to the tendency for evolutionary differentiation among species. During evolution, both the oat and rice *GRAS* genes were derived from the same ancestor.

The promoter region of AsGRAS family genes contains numerous *cis-*elements, which can be divided into three categories according to their action mechanisms: light-responsive elements, stress-responsive elements, and hormone-responsive elements. However, the types and numbers of elements in different subfamily members are quite different, indicating that different members respond to different light conditions. Various kinds of light-responsive elements were also found in the promoter regions of other species, such as *O. sativa* (Dutta et al., 2021), *T. aestivum* (He et al., 2021), and *G. max* (Wang et al., 2020a). Photoinduced *OsGRAS* gene expression in *O. sativa* was found at the transcriptional level (Liu & Widmer, 2014), indicating that AsGRAS family genes were probably also involved in the light response process. Numerous experimental results showed that the expression of each member of the plant GRAS family gene was induced by stress factors such as high temperature, low temperature, and drought (Zeng et al., 2019; Laskar et al., 2021; Wang et al., 2020b). Our study showed that the promoters of the AsGRAS gene family had a low-temperature response element, a drought-inducible response element, and defense and stress response elements. The results of this study indicate that oat AsGRAS family genes may be involved in various environmental stress responses and may improve oat tolerance to adversity. The AsGRAS family of genes has different hormone response elements in their promoters, including abscisic acid, gibberellin, salicylic acid, methyl jasmonate, and other response elements. Prior studies have demonstrated that hormones may also act as a class of trans-acting factors that may bind to hormone-responsive elements on the promoters to regulate the transcriptional expression of target genes (Mundy, Yamaguchi-Shinozaki & Chua, 1990). In addition to their direct involvement in organismal growth and development, hormones also function as signal transduction factors (such as ABA) to participate in stress-response processes such as low-temperature, high-temperature, and salt stress (Yusuf et al., 2013; Gunes et al., 2007). Thus, we can hypothesize that the expression of AsGRAS family genes in oat may be hormone-induced and participate in the scavenging of excess reactive oxygen species in the presence of stress, thereby promoting the antioxidant function of oat plants.

Several types of *GRAS* genes in plants (for example, *Cucumis sativus*, *Panicum virgatum*, *Setaria italica*, *Manihot esculenta*, and *Medicago sativa*) have been reported to play a role in abiotic stress responses (Lu et al., 2020; Wang et al., 2021; Fan et al., 2021; Shan et al., 2020; Zhang et al., 2021b). With the aim of exploring the expression patterns of oat *GRAS* genes in different tissues subjected to different abiotic stresses, we used qRT-PCR to analyze the *AsGRAS* gene family expression profiles in this study. Although the results showed that the oat SCR, PAT1, HAM, and DELLA subfamilies were responsive to abiotic stress, the PAT1 subfamily and HAM subfamily were the most involved as they had the largest number *AsGRAS* genes in oat. Prior studies have revealed that the GRAS gene family has essential functions in the stress response of various plants, which is important for future molecular breeding innovations (Cenci & Rouard, 2017). The *GRAS* genes *VviPAT3*, *VviPAT4*, *VviPAT6*, and *VviPAT7* from the PAT subfamily in *V. vinifera* all have higher expression levels in the presence of drought stress at the seedling stage (Grimplet et al., 2016). Members of the HAM subfamily play important roles in the abiotic stress response of *S. lycopersicum* (Huang et al., 2015b). *OsGRAS23* expression was increased under drought-stress conditions. Likewise, *OsGRAS2*, a prominent member of the GRAS family of *O. sativa*, is also responsive to the drought stress mechanism of *O. sativa* (Xu et al., 2015). Thus, to identify the *AsGRAS* genes in oat that have functions related to drought and salt stress responses, we performed a preliminary screen for genes whose expression was upregulated under drought and salt treatments. We found that the expression levels of *AsGRAS01*, *AsGRAS12*, *AsGRAS14*, and *AsGRAS21* in the HAM subfamily and *AsGRAS24* in the PAT1 subfamily were all upregulated. *AsGRAS12* and *AsGRAS21* expression increased in leaf tissue after 6 h of drought stress, while the others began to increase after 3 h of drought stress. The expression levels of both *AsGRAS01* and *AsGRAS12* increased in root tissue following 3 h of drought stress. Other genes showed similar expression patterns in the root tissue, with the highest expression level coming after 6 h of drought stress. In this study, we further confirmed that the GRAS gene family was essential to the abiotic stress response.

## Conclusions

In this study, we identified 30 *AsGRAS* genes in oat, which were unevenly distributed across chromosomes in the oat genome. The identified *AsGRAS* members were further divided into four GRAS subfamilies: SCR, PAT1, HAM, and DELLA. Most of the *AsGRAS* genes lacked introns, indicating that the *AsGRAS* gene structure was highly conserved. The *AsGRAS* members of the same subfamily or branch shared extensive similarities, suggesting that they have similar gene functions. The qRT-PCR analysis identified many *AsGRAS* genes (*AsGRAS01*, *AsGRAS12*, *AsGRAS14*, *AsGRAS21*, and *AsGRAS24*) with potential regulatory roles for drought stress and osmotic stress, and these genes may become the focus of future studies. However, the current study only provides a preliminary *AsGRAS* gene signature in oat plants and further functional validation is needed to understand the role of *AsGRAS* genes in different biological processes.

## Supplemental Information

10.7717/peerj.15370/supp-1 Supplemental Information 1 Sequences of the primers used in this study.

10.7717/peerj.15370/supp-2 Supplemental Information 2 The 30 identified *AsGRAS* genes in this study.

10.7717/peerj.15370/supp-3 Supplemental Information 3 Corresponding names of 32 *AtGRAS* genes in *Arabidopsis*.

10.7717/peerj.15370/supp-4 Supplemental Information 4 Gene ID of GRAS gene family members from four model plants: *Arabidopsis thaliana, Oryza sativa, Zea mays and Avena sativa*.

10.7717/peerj.15370/supp-5 Supplemental Information 5 One-to-one orthologous relationships between the GRAS gene members in Avena Sativa and other species.

10.7717/peerj.15370/supp-6 Supplemental Information 6 Cis-element analyses of the *AsGRAS* gene promoter regions.

10.7717/peerj.15370/supp-7 Supplemental Information 7 Expression profiles of *AsGRAS* genes in oat leaf and root during drought and salt stresses. The raw data of the qRT-PCR results.

## Additional Information and Declarations

### Data Availability

The following information was supplied regarding data availability: The raw data are available in the Supplemental Files.
